# Aerobic fitness in professional soccer players after anterior cruciate ligament reconstruction

**DOI:** 10.1371/journal.pone.0194432

**Published:** 2018-03-22

**Authors:** Adriano Marques de Almeida, Paulo Roberto Santos Silva, André Pedrinelli, Arnaldo J. Hernandez

**Affiliations:** Sports Medicine Discipline, Instituto de Ortopedia e Traumatologia, Hospital das Clínicas HCFMUSP, Faculdade de Medicina, Universidade de Sao Paulo, Sao Paulo, SP, Brazil; University of L'Aquila, ITALY

## Abstract

Although anterior cruciate ligament (ACL) reconstruction is considered a successful procedure in restoring knee stability, few studies have addressed the issue of aerobic capacity after ACL surgery. Soccer players need technical, tactical and physical skills to succeed, such as good knee function and aerobic capacity. Our purpose is to evaluate aerobic fitness in ACL injured professional football players and six months after ACL reconstruction compared to a control group. Twenty athletes with ACL injury were evaluated and underwent ACL reconstruction with hamstrings autograft, and were compared to twenty healthy professional soccer players. The methods used to evaluate aerobic fitness were maximum oxygen uptake (VO_2max_) and ventilatory thresholds with a treadmill protocol, before and six months after surgery, compared to a control group. Knee function questionnaires, isokinetic strength testing and body composition evaluation were also performed. Results: Median ACL-injured patients age was 21 years old, and controls 20.5 years old. (n.s.). Preoperative VO_2max_ in the ACL injured group was 45.2 ± 4.3 mL/kg/min, postoperative 48.9 ± 3.8 mL/kg/min and controls 56.9 ± 4.2 mL/kg/min. (p< .001 in all comparisons). Body composition evaluation was similar in all situations. Knee function questionnaires and quadriceps peak torque deficit improved after surgery but were significantly lower compared to controls. Conclusion: Aerobic fitness is significantly reduced in professional soccer players with ACL injury, and six months of rehabilitation was not enough to restore aerobic function after ACL reconstruction, compared to non-injured players of the same level.

## Introduction

Anterior cruciate ligament (ACL) injury is one of the most frequent severe knee injuries in soccer players.[1] The advances in surgical techniques have resulted in good surgical outcomes and increased the potential for an athlete to return to his previous level of sport activity.[2] Many studies outline criteria to successfully return to sport with minimum risk of reinjury. These criteria include time after ACL reconstruction (ACLR), knee stability, isokinetic strength tests, hop tests and self-reported questionnaires. [3–5] Although most studies list six months as the time period allowed for return to sports[6], it has been shown that most athletes do not meet these criteria at this period[4,5], and up to 45% of participants do not return to their preinjury level of sports participation.[7]

Soccer players need technical, tactical and physical skills to succeed, including high endurance capacity and strength.[8,9] During a 90-minute game, soccer players run about 10km at an average intensity close to the anaerobic threshold.[8] Maximum oxygen uptake (VO_2max_) is defined as the highest rate at which oxygen can be taken up and utilized by the body during severe exercise. It is the most common method to evaluate cardiorespiratory fitness of an individual. [10,11] A VO_2max_ of 60 ml/kg/min has been suggested as the minimum fitness requirement for male soccer players to play at elite level. [8,9,12] However, few studies have addressed the issue of aerobic capacity of professional soccer players after ACL reconstruction. [13–15]

The purpose of this study was to evaluate aerobic fitness in ACL injured professional soccer players, before and six months after ACL reconstruction, compared to a control group of non-injured professional soccer players. A secondary aim was to evaluate ventilatory thresholds and running speeds, body composition, isokinetic knee strength and self-reported knee function questionnaires.

## Materials and methods

This was a prospective case-control study to evaluate aerobic fitness of professional soccer players with ACL injury and after ACL reconstruction, compared to professional players without knee injury.

This study took place at the Sports Medicine Department of University of São Paulo. This study followed the ethical statements of the Declaration of Helsinki and was approved by the University of São Paulo Medical School Institutional Review Board (protocol 368105) and registered in a public registry (NCT02674282). Written informed consents explaining the study objectives, procedures, benefits and risks were obtained from all participants. The inclusion criteria were ACL injury, professional outfield soccer player, male gender, absence of open physes. Exclusion criteria were associated ligament lesions requiring surgical repair or reconstruction, knee effusion, impossibility to perform the running test, previous knee surgeries, post-operative infection, arthrofibrosis, ACL reconstruction failure, inadequate follow-up. Inclusion criteria for controls were the same, except for ACL injury, or any previous injury that required surgical treatment.

In total, 29 ACL injured patients met the inclusion criteria. Four patients declined to participate. One patient had a bucket-handle meniscus injury and was unable to complete the running test. Twenty-five patients were enrolled to the study. Four ACL injured patients were lost to follow-up, and one patient was excluded due to a cyclops lesion that needed a reoperation during the study. Therefore 20 patients completed the study. The median time between injury and surgery was 3 months (range 1 to 12 months). The control group consisted of 20 soccer players from local lower division professional soccer teams that were performing routine evaluations at our facility.

The measurements were performed prior to the surgery and six months after ACL reconstruction in the ACL group, and were compared to their pre-operative values, as well as to a control group.

### Cardiopulmonary exercise testing

The primary outcome of this study was the maximum oxygen uptake (VO_2max_) of professional soccer players six months after ACL reconstruction, compared to pre-operative and controls results. Cardiopulmonary exercise test on a treadmill was used to evaluate VO_2max_ and ventilatory thresholds with a modified Heck protocol.[11]

The tests were performed in the afternoon, starting at 01:00 PM, in our laboratory, at room temperature ranging from 20°C to 24°C, relative air humidity between 45% and 65% and barometric pressures from 698 mmHg to 705 mmHg. The athletes were advised to avoid high intensity exercises during the 24 hours preceding the test and not to have drinks with caffeine or alcohol on the evaluation day. They had a light meal 1 to 2 hours before the test and came for the test dressed in T-shirts, shorts and running shoes.

An electrocardiogram with 13 derivations (Heartware, Ergo13, Belo Horizonte, Brazil) was recorded during rest, effort and recovery phase. Arterial blood pressure was measured indirectly using an aneroid sphygmomanometer (Tycos, USA) at the end of each stage of the test. The equation 208 –(0.7 x age) was used to find maximal heart rate.[16]

Cardiopulmonary exercise testing using a modified Heck protocol was performed on a motorized treadmill (h/p/cosmos®, Pulsar, Germany). The test began at 8.4 km/h, with increments of 1.2 km/h every two minutes. The inclination was fixed at 2%. The quantity of oxygen (O_2_) consumed, carbon dioxide (CO_2_) produced and pulmonary ventilation (V_E_) were measured breath-by-breath with a computerized gas exchange analysis system (CPX/ULTIMA™, MGC Diagnostics®, Saint Paul, MN, USA) calibrated before each test. The data was analyzed with BreezeSuite™ 6.4.1 cardiorespiratory diagnostic software (MGC Diagnostics®, Saint Paul, MN, USA).

We considered the test had reached its maximum when the players attained 2 of the following criteria: 1) respiratory exchange ratio (VCO_2_/VO_2_) ≥ 1.10; 2) Borg’s scale of subjective perception of tiredness ≥ 18 and 3) a constant heart rate (HR) varying within 5 beats/min of the predicted. The VO_2max_ was quantified by one of the authors (PRSS), an experienced sports physiologist, when the VO_2max_ increased less than 2 mL/kg/min between two consecutive stages in the peak of the exercise. We also determined two ventilatory thresholds (VTs) during the test.[17] The first ventilatory threshold (VT_1_) was determined when we observed the lowest ventilatory equivalent of oxygen (V_E_/VO_2_) before its continuous increase, and the lowest end-tidal oxygen tension (PETO_2_) before its continuous increase. The second ventilatory threshold (VT_2_) was determined when we observed the lowest ventilatory equivalent of carbon dioxide (V_E_/VCO_2_) before its continuous increase and the highest end-tidal carbon dioxide tension (PETO_2_) preceding its abrupt fall (second inflection of curves in progressive exercise). This is the transition between “steady” and “heavy” paces. We also determined the running speed and the percentage of VO_2max_ in which the VT_2_ occurred.

### Body composition, isokinetic strength and knee function evaluations

Body composition evaluation was performed before the running test. We used a tetrapolar bioelectrical impedance analyzer (InBody 230, Seoul, Corea) to measure weight, fat-free mass (FFM), body fat (BF) and body mass index (BMI). The evaluation was performed in the morning, before lunch. We measured height and oriented individuals to void before the test.[18]

Strength measurement of knee extensors and flexors muscles were performed using an isokinetic dynamometer (Biodex System 3 Pro, Biodex Inc. NY, USA). The subjects performed a 5-minute warm-up on a cycloergometer before the evaluations were conducted. The test consisted of a series of five maximal concentric flexion and extension movements at a constant angular velocity of 60°/s and twenty flexion and extension movements at a constant angular velocity of 240°/s. Peak torque of knee extensors and knee flexors muscles were normalized by body mass. We also quantified the peak torque deficits comparing the injured and non-injured limbs.[19]

Participants completed self-reported knee function questionnaires Lysholm and International Knee Documentation Committee (IKDC) Subjective Knee Evaluation Form, both validated to use in the Brazilian population. [20,21]

### Surgical procedure

A single surgeon performed all ACL reconstructions with the same surgical technique. Cefazolin (1g via the intravenous route) was administered prophylactically. Patients were positioned supine in the operating table. Under regional anesthesia, air tourniquet was applied to the proximal thigh with a pressure of 350mmHg. A longitudinal 3 to 4 cm incision was made in the anteromedial aspect of the proximal leg. The semitendinosus and gracilis tendons were identified, released from their insertions on the tibia and harvested with a tendon stripper. The tendons were cleaned of soft tissue and folded to prepare a 4-stranded graft. Anatomic ACL reconstruction was performed arthroscopically. The femoral tunnel was drilled inside out through the anteromedial portal in the ACL footprint.[22] Femoral fixation was performed with a continuous loop fixed-length cortical suspension device (endobutton)[23] for all patients. The tibial tunnel was drilled outside in at the center of the ACL footprint and the graft was fixed within the tibia with metallic interference screw.

### Statistical analysis

A pilot study was performed *a priori* with six soccer players with ACL injury. In this group VO_2max_ was 41.91 ± 5.59 ml/kg/min. Based on previous studies [13,24,25] we considered that a VO_2max_ variation of 15% would be clinically significant, and calculated the sample size for a level of significance of .05 and power of .90, resulting in 20 subjects per group.

Descriptive statistics was performed with mean and standard deviation for continuous data, and median and range for ordinal data. Inferential statistics comparing ACL and ACLR values were performed with Student’s paired t-test or Wilcoxon test, and comparing the ACL or ACLR with the control group with Student’s t-test or Mann-Whitney U-test. We considered the level of significance p < .05.

## Results

The groups were similar regarding to age. Body composition evaluation showed no significant differences in the ACL group comparing the preoperative with the postoperative moments, neither in the comparison with the control group (Table 1).

**Table 1:** Baseline and body composition measurements of athletes with ACL injury, after ACLR and controls.

	ACL	ACLR	Control	ACL vs ACLR	ACL vs control	ACLR vs control
					p value	
Age, years median (range)	21 (18–28)	-	20.5 (18–34)	-	.99	-
Weight (kg)	79.2 ± 10.1	79.3 ± 8.9	74.8 ± 6.2	.20	.12	.07
Height (m)	1.82 ± 0.08	-	1.79 ± 0.07	-	.13	-
Fat-free mass (kg)	67.2 ± 8.0	67.3 ± 6.8	65.2 ± 6.1	.34	.38	.32
Body fat (%)	14.7 ± 3.7	14.9 ± 5.4	12.8 ± 4.0	.76	.12	.16
Muscle mass (kg)	38.4 ± 4.8	38.4 ± 4.1	37.3 ± 3.6	.36	.43	.37
BMI	23.7 ± 2.0	24.0 ± 2.0	23.4 ± 1.8	.11	.68	.34

BMI: body mass index

VO_2max_ in the control group was 56.9 ± 4.2 mL/kg/min. In the athletes with ACL injury VO_2max_ was 45.2 ± 4.3 and after ACLR increased to 48.9 ± 3.8. All comparisons were statistically significant (p< .001). VT_1_ and VT_2_ were also lower after ACLR than in the control group, but significantly higher than before surgery (Table 2). Average ACL injured and ACLR VO_2max_ values were 20% and 14% lower than controls, respectively. All comparisons were statistically significant. In the ACL injured athletes, the correlation between time from injury and Vo_2max_ was not significant, neither at the pre-operative period (r_s_ = .064, p = .79), nor at the post-operative period (r_s_ = -.24, p = .3).

**Table 2:** VO_2max_ and ventilatory thresholds before and after ACLR and in the control group.

	ACL	ACLR	Control	ACL vs ACLR	ACL vs control	ACLR vs control
				p value [IC 95% of the difference]		
VO_2max_ (mL/kg/min)	45.2 ± 4.3	48.9 ± 3.8	56.9 ± 4.2	< .001 [-5.6, -2.0]	< .001 [8.9, 14.4]	< .001 [5.4, 10.5]
vVO_2max_ (km/h)	14.4 (10.8–19.2)	15.6 (13.2–19.2)	16.8 (15.6–19.2)	.007 [1.8, 3.8]	< .001 [1.8, 3.8]	< .001 [0.9, 2.7]
VT_2_ (mL/kg/min)	38.3 ± 4.1	41.4 ± 4.5	49.1 ± 3.6	.008 [-5.5, -0.9]	< .001 [8.3, 13.2]	< .001 [5.0, 10.2]
%VO_2max_VT_2_	85 ± 6	85 ± 7	86 ± 4	.92 [-4.8, 5.3]	.39 [-1.9, 4.9]	.36 [-2.1, 5.6]
vVT_2_ (km/h)	10.8 (9.6–13.2)	12 (10.8–13.2)	13.2 (12.0–15.6)	.006 [1.7, 2.9]	< .001 [1.7, 2.9]	< .001 [1.1, 2.3]
VT_1_ (mL/kg/min)	30.3 ± 5.1	34.3 ± 3.5	37.2 ± 3.7	.006 [-6.7, -1.3]	< .001 [4.1, 9.8]	.015 [0.6, 5.3]
%VO_2max_VT_1_	67 ± 8	70 ± 7	65 ± 6	.22 [-9, 2.2]	.61 [-6.1, 3.6]	.04 [-9.1, -0.2]
vVT_1_ (km·h^-1^)	9.6 (7.2–10.8)	9.6 (8.4–10.8)	9.6 (9.6–12.0)	.025 [0.5, 1.5]	< .001 [0.5, 1.5]	.095 [-0.4, 0.9]

VO_2max_: maximum oxygen uptake; VT_1_ and VT_2_: first and second ventilatory thresholds

%VO_2max_VT_1_ and %VO_2max_VT_2_: percentage of VO_2max_ corresponding to VT_1_ and VT_2_

vVO_2max_, vVT_1_ and vVT_2:_ running speeds at VO_2max_, VT_1_ and VT_2_, median (range).All other values represent mean ± standard deviation

Running speed at VO_2max_, at VT_1_ and at VT_2_ were significantly lower in the ACL group than in the control group (Table 2). It increased after ACLR, although still lower than controls. All comparisons were statistically significant, except for the running speed at VT_1_ comparing the ACLR with the control group. The percentage of respective VO_2max_ at which the ventilatory thresholds occurred were similar in all situations, except for the VT_1_ comparing ACLR and controls.

Median knee function score IKDC improved from 59.2 to 90.8 (p< .001), compared to 100 in the control group (p< .001) and median Lysholm score improved from 80 to 95 (p< .001), compared to 100 in the control group (p = .03) (Table 3). Average extension peak torque deficit at 60^o^s after ACLR was 15.7%. The comparison between ACLR and the control group showed a statistically significant difference in the extensor and flexor peak torque normalized by body mass and peak torque deficit.

**Table 3:** Self-reported knee function questionnaire results, median (range), and isokinetic knee extensors and flexors strength at 60 ^o^/s, normalized by body mass (mean and standard deviation).

	ACL	ACLR	Control	ACL vs ACLR	ACL vs control	ACLR vs control
					p value	
IKDC	59.2 (26.4–90.8)	90.8 (63–100)	100 (86–100)	< .001	< .001	< .001
Lysholm	80 (39–95)	95 (77–100)	100 (90–100)	< .001	< .001	.030
Extension (N.m/Kg)	252.2 ± 60.6	291.3 ± 45.5	358 ± 44.2	.008	< .001	< .001
Extension PT deficit (%)	21.5 ± 18.6	15.7 ± 13.2	3.1 ± 13.9	.630	< .001	< .001
Flexion (N.m/Kg)	151.2 ± 34.3	166.1 ± 30.9	190.5 ± 18.5	.023	< .001	.005
Flexion PT deficit (%)	7.2 ± 17.0	10.7 ± 14.6	2.0 ± 8.0	.410	.231	.026

IKDC: International Knee Documentation Committee Subjective Knee Evaluation Form; PT deficit: peak torque deficit

## Discussion

ACL tears are severe injuries, and may impact the career of a professional soccer player.[26] Surgical treatment leads to a phase of reduced activity and physical deconditioning.[13] However, few studies addressed the issue of aerobic fitness after ACLR in soccer players. In this study, we showed that VO_2max_ is highly reduced in ACL injured professional soccer players and, although it shows an improvement six months after ACLR and rehabilitation, it is still significantly lower than we observed in players of the same level.

VO_2max_ is one of the most important parameters for endurance performance, and has been extensively assessed among soccer players, [8,11,25,27] although not after ACLR. Soccer demands a high level of aerobic fitness to be successfully played at a professional level. A VO_2max_ of 60 ml/kg/min has been suggested as the minimum fitness requirement to play at the men’s professional level.[25] Data from 1545 Norwegian male soccer players indicate that VO_2max_ values from 62 to 64 ml/kg/min fulfill the demands in men’s professional soccer, although players from third to fifth division teams demonstrated an average 2 mL/kg/min lower VO_2max_ than the higher divisions players.[12] In our study, control group athletes showed a 56.8 ± 4.2 mL/kg/min VO_2max_. These VO_2max_ values were significantly higher than the values we found six months after ACLR, 48.9 ± 3.8 mL/kg/min, and higher than we found before surgery, 45.2 ± 4.3, which represents a 20% lower VO_2max_ when compared to the control group. For comparison purposes, the age-related decline in VO_2max_ is reported at 10% per decade.[28] Obviously, endurance is not the only factor related to soccer performance, and technical and tactical skills may make up for a fitness deficiency, but some studies show that the VO_2max_ is directly related to soccer performance. [24] Also, it has been demonstrated that VO_2max_ is an independent predictor of injury in soccer players. [29]

VO_2max_ may be affected by the lung capacity, cardiac output and O_2_ carrying capacity of the blood, termed central factors, and by the skeletal muscle efficiency, termed peripheral factor. Accordingly, to the equation of Fick, VO_2_ = cardiac output x arteriovenous oxygen difference. Cardiac output is reduced after knee surgery in soccer players.[13] Skeletal muscle O_2_ extraction is also reduced with the reduction in muscle power and strength. Although both factors are probably involved, previous studies show that central factors are viewed as the main limiting factor for VO_2max_.[10]

VT_1_ represents the anaerobic threshold measured by ventilatory changes [11,30] and is a predictor of endurance capacity considered more sensitive to the training status than VO_2max_.[31] Because of the game duration, soccer is mainly dependent upon aerobic metabolism, but soccer matches show periods and situations of high intensity where the anaerobic metabolism prevails.[8] The reduction in VT_1_ can be explained by a worse ATP turnover through the aerobic pathway and rapid involvement of the glycolytic pathway.[32] The VT_2,_ or respiratory compensation point, is related to the acid-basis regulation and the efficiency of the buffer system to keep pH in the blood and peripheral muscle. Rehabilitation exercises improves the buffer system in the muscle[33] explaining the improvement in VT_2_ after rehabilitation. The improvement in VT_1_ and VT_2_ observed in ACL patients after reconstruction indicates a better physical conditioning after surgery than before. However, all results were lower than observed in the control group.

There is a possibility that the poor aerobic fitness observed in the ACL injured players contributed to the injury itself. To evaluate this hypothesis, we would need the baseline results of these athletes. This is an interesting field for research, and some papers are finding results to corroborate this. [29] In this case, it reinforces the need to evaluate and improve the aerobic fitness of professional soccer players before they return to play, to avoid other injuries.

ACL surgery is considered effective in restoring knee function, and also to allow the participation in high demand activities such as professional soccer.[34,35] However, the concept of studying athletic performance on return to sport from ACL reconstruction is relatively new and under-researched.[36] We chose the six-month follow-up period to perform postoperative evaluation because this is usually the period we are considering to allow the athlete to return to play.[6] The patients performed the test at six months, independently of other criteria for return to play. In this study we found a postoperative median IKDC subjective score of 90.8 and median Lysholm score of 95, which are considered excellent results.[20,21]. Also, we observed a postoperative quadriceps strength deficit of 15.7%, which is in accordance with the literature.[37,38] Moreover, body composition measurements in the ACL athletes and after ACLR showed no significant differences compared to controls. Despite these satisfactory results[5,6], recent evidence show that up to 45% of athletes do not return to their previous level of sports activity.[7] The deficit in VO_2max_ observed in this study may be one additional factor that help explain the discrepancy between knee function recovery and sports performance after ACL reconstruction. [13–15,39] At the best of our knowledge, no previous study addressed the VO_2max_ after ACLR rehabilitation as the primary outcome measure. [5,15]

This is not a randomized study, and therefore many forms of bias, including selection bias, may occur. We tried to avoid this by choosing a control group that matched our patients, all of them being professional soccer players at the second and third state divisions. The control group was tested only once, during their competitive season, and were not prospectively followed, as the ACL injured athletes. Since that no intervention was performed in the control group, we did not expect a significant time effect during the six months in this group, although it might occur. Our baseline and anthropometric data show that the samples are comparable in terms of age and body composition. Second, one may argue that the treadmill test could be jeopardized by the knee condition of the individual, resulting in a VO_2max_ not representative of the actual aerobic fitness. However, during the tests, all athletes reached the criteria for a maximal test, defined in the methods session. Only one patient, with a bucket-handle meniscal injury, was unable to conclude the test because of knee symptoms. Moreover, it is known that the VO_2max_ obtained with cycle ergometer and bicycle protocols are lower than those obtained with treadmill testing.[8] Also, soccer players should use the treadmill as this mode of exercise is closer to their specific activity. Third, the three-month time span between injury and surgery may be considered too long, mainly in professional players. This occurred because of the characteristic of our public health system, although we don’t think this period is excessive. Also, it allowed us to evaluate the effect of ACL injury on deconditioning prior to ACLR.

Therefore, VO_2max_ is significantly reduced in soccer players with ACL injury, and six months of standard rehabilitation was not enough to restore aerobic function after ACL reconstruction. More attention should be paid to aerobic conditioning in professional soccer players at return to sports after ACLR, and aerobic function evaluation should be considered in high level athletes. These findings should have implications for the routine rehabilitation after ACL reconstruction in soccer players. Future research should focus on strategies to prevent aerobic function deconditioning in athletes with ACL injury before and after surgery.

## Conclusions

We conclude that the aerobic capacity of professional soccer players six months after ACL reconstruction, measured by VO_2max,_ ventilatory thresholds and running speeds, are significantly lower than in the control group athletes, although higher than before surgery. The knee function scores showed a significant improvement after surgery, and body composition evaluations were not significantly different.

## Supporting information

S1 File VO_2max_ and ventilatory thresholds of ACL injured athletes and healthy controls. (XLSX)

S2 File Body composition evaluation of ACL injured athletes and healthy controls. (XLSX)
